# 587 Ambient temperatures in the burn operating room: A QI initiative

**DOI:** 10.1093/jbcr/irac012.215

**Published:** 2022-03-23

**Authors:** Alan D Rogers, Robert Cartotto, David L Wallace, Syena Moltaji

**Affiliations:** Ross Tilley Burn Centre, Toronto, Ontario; Ross Tilley Burn Centre, Sunnybrook HSC, Toronto, Toronto, Ontario; University of California, Davis, Sacramento, California; Ross Tilley Burn Centre, Sunnybrook HSC and Division of Plastic and Reconstructive Surgery, University of Toronto, Toronto, Ontario

## Abstract

**Introduction:**

Patients with burn injuries are particularly susceptible to perioperative hypothermia, which is associated with a range of complications, including infection, bleeding and delayed wound healing. Raising the operating room (OR) ambient temperature is an important strategy to prevent hypothermia, but we were uncertain about how strictly we do this. The purpose of this study was to determine the ambient OR temperatures during acute burn surgery as the first step of a quality improvement initiative to ensure we are maintaining a warm OR environment.

**Methods:**

Between 1/3/2020 and 28/2/2021, ambient temperatures during surgery in the burn OR of an ABA-verified burn center were recorded every 15 minutes. Temperatures were measured using a wall-mounted smart sensor, transmitted to a mobile smartphone application via Bluetooth. All patients undergoing acute burn excision and closure procedures lasting > 2 hours were included in the study. Hypothermia was defined as a core temperature < 36°C.

**Results:**

Of the 119 patients, the majority were male (n=86; 72.3%), the mean age was 49.8 years (range 18-89) and the mean total body surface area (TBSA) was 15.8% (range 1-62%). They underwent 261 operations (mean 2.2 cases per patient; range 1-15) with a mean duration of 211.1 minutes (range 120-468 minutes). Sixty-two patients underwent 1 surgery, and 31 had 2 surgeries. Eight patients (6.7%) died. Thirty-three patients (27.7%) were hypothermic at the end of 43 cases (16.5%).

The mean ambient temperature was 24.8^o^C (range 18.7^o^C to 29.6^o^C). Hypothermic patients (n= 33 patients, 43 cases), did not differ significantly in age or operative duration from non- hypothermic patients (n= 86 patients, 218 cases) but did have significantly larger %TBSA burns [32.3 vs 24.8 (p= 0.02)]. Hypothermic cases experienced significantly lower ambient temperatures at the start of surgery (p=0.007), and mean temperature during surgery (p=0.009), and tended to experience a lower ambient temperature at the end of surgery (24.5 C vs 25.1 °C , p= 0.06). [Table]

**Conclusions:**

We have identified that the ambient temperature in our burn OR is lower than desirable and that this is directly related to development of hypothermia. These results now indicate that a QI intervention (stricter attention and manoeuvers to ensuring a warm operating room at the start and during surgery) is required.

